# Efficacy of a Russian-backbone live attenuated influenza vaccine among children in Senegal: a randomised, double-blind, placebo-controlled trial

**DOI:** 10.1016/S2214-109X(16)30201-7

**Published:** 2016-10-13

**Authors:** John C Victor, Kristen D C Lewis, Aldiouma Diallo, Mbayame N Niang, Bou Diarra, Ndongo Dia, Justin R Ortiz, Marc-Alain Widdowson, Jodi Feser, Rebecca Hoagland, Shannon L Emery, Kathryn E Lafond, Kathleen M Neuzil

**Affiliations:** a PATH, Seattle, WA, USA; b Mixed Research Unit 198, Institut de Recherche Pour le Développement, Dakar, Senegal; c Senegal National Influenza Center, Institut Pasteur de Dakar, Dakar, Senegal; d Departments of Global Health and Medicine, University of Washington, Seattle, WA, USA; e Influenza Division, National Center for Immunization and Respiratory Diseases, Centers for Disease Control and Prevention, Atlanta, GA, USA; f Cota Enterprises Inc, McLouth, KS, USA; g Center for Vaccine Development, University of Maryland School of Medicine, Baltimore, MD, USA

## Abstract

**Background:**

Live attenuated influenza vaccines have been shown to significantly reduce influenza in diverse populations of children, but no efficacy studies have been done in resource-poor tropical settings. In Senegal, we assessed the efficacy and safety of a live attenuated influenza vaccine based on Russian-derived master donor viruses and licensed as a single dose.

**Methods:**

In this double-blind, placebo-controlled, parallel group, single-centre trial done near Niakhar, Senegal, generally healthy children aged 2–5 years were randomly allocated (2:1) to receive a single intranasal dose of masked trivalent live attenuated influenza vaccine or placebo. The allocation sequence was computer-generated by PATH with block sizes of three. The manufacturer provided vaccine and placebo in coded vials to preserve blinding. Participants were monitored through the predictable influenza season in Senegal for adverse events and signs and symptoms of influenza using weekly home visits and surveillance in clinics. The primary outcome was symptomatic laboratory-confirmed influenza caused by any strain and occurring from 15 days post-vaccination to the end of the study. The primary analysis was per protocol. This study is registered with ClinicalTrials.gov, number NCT01854632.

**Findings:**

Between May 23, and July 1, 2013, 1761 children were randomly assigned, 1174 to receive live attenuated influenza vaccine and 587 to receive placebo. The per-protocol set included 1173 vaccinees and 584 placebo recipients followed up to Dec 20, 2013. Symptomatic influenza was laboratory-confirmed in 210 (18%) of 1173 recipients of live attenuated influenza vaccine and 105 (18%) of placebo recipients, giving a vaccine efficacy of 0·0% (95% CI −26·4 to 20·9). Adverse events were balanced between the study groups. Two girls who had received live attenuated influenza vaccine died, one due to anasarca 12 days postvaccination and one due to malnutrition 70 days postvaccination.

**Interpretation:**

Live attenuated influenza vaccine was well tolerated in young children in Senegal, but did not provide protection against influenza. Further study in such populations, which might experience extended periods of influenza circulation, is warranted.

**Funding:**

US Centers for Disease Control and Prevention and Bill & Melinda Gates Foundation.

## Introduction

Globally, lower respiratory infection remains the leading cause of death in children younger than 5 years,^1^, ^2^, ^3^ and influenza virus infections can be an important contributor to serious lower respiratory disease.^4^ Live attenuated influenza vaccines are an attractive option for young children in low-resource settings. In US trials done before this trial was initiated, live attenuated influenza vaccines had shown superior efficacy to inactivated vaccines among young children and might be cross-protective against drifted influenza viruses.^5^, ^6^ Live attenuated influenza vaccines are likewise attractive from a manufacturing perspective because higher yields, a simpler purification process, and quicker lot release should lower the cost of production of live attenuated influenza vaccines relative to inactivated vaccines.^7^ Finally, live attenuated influenza vaccines might be more feasible given their intranasal administration and potential protection with a single dose.^8^

In Senegal, there are no recommendations for routine influenza vaccination of children, and generally only trivalent inactivated influenza vaccine of WHO-recommended northern hemisphere formulation has been available in the country for use in vaccination of pilgrims to the Hajj. The National Influenza Center of Senegal has been undertaking influenza surveillance for more than 20 years and has accumulated data that influenza, especially type A, occurs in association with the rainy season^9^ (typically between July and September) and can circulate widely, especially among children.

Under the Global Pandemic Influenza Action Plan to Increase Vaccine Supply, the WHO leads a programme to enhance regional access to influenza vaccines by supporting developing country vaccine manufacturers.^10^ Serum Institute of India, Ltd (Pune, India) participated in this initiative and developed a reassortant live attenuated seasonal influenza vaccine based on A/Leningrad/17 and B/USSR/60 master donor viruses. To understand how live attenuated influenza vaccines such as this might perform in young children in resource-limited tropical settings in Africa, we assessed the efficacy of Serum Institute of India's trivalent live attenuated influenza vaccine, which is licensed as a single dose for prevention of influenza, in Senegal.

**Research in context**

**Evidence before this study**

We searched PubMed from Jan 1, 1980, to Jan 1, 2016, for efficacy trials assessing protection by live attenuated influenza vaccines among children using the search terms, “human influenza”, “vaccines, attenuated”, and “children”. Live attenuated influenza vaccines have been studied mainly in developed countries in Europe, Asia, and in the USA, but one study has included children aged 6 months to 36 months of age from sites in South Africa. In nearly all studies, A/Ann Arbor-based live attenuated influenza vaccines provided significant protection. Russian-derived live attenuated influenza vaccines used among young children had been studied primarily from 1986 to 1991 in five trials in the Soviet Union and Cuba. These studies used serological confirmation of illness and were done before introduction of Good Clinical Practice standards for the designing, conducting, recording, and reporting of trials. Moreover, except for the companion study in Bangladesh, no efficacy trials of Russian-derived live attenuated influenza vaccines have been done in developing country settings. The only study of clinical protection of Serum Institute of India's live attenuated influenza vaccines was an observational case-control study of the effectiveness of one dose of its monovalent live attenuated influenza vaccines containing vaccine virus antigenically similar to pandemic A/H1N1 (2009).

**Added value of this study**

To the best of our knowledge, our trial is the first randomised trial of any live attenuated influenza vaccines in tropical developing Africa, and our results indicate that children in this population were not protected with one dose. Our results point to the need for more study of live attenuated influenza vaccines in impoverished populations in Africa to understand how well live attenuated influenza vaccines do and can best be used to provide protection to young children.

**Implications of all the available evidence**

The results of this study should be viewed in light of data from early studies in developed populations and the results of our companion study in Asia in which live attenuated influenza vaccines provided moderately high protection to this young child age group. Counter to this finding is the fact that observational effectiveness studies in the USA in 2013–14 found a similar absence of efficacy of live attenuated influenza vaccines against A/H1N1 (2009) in young children. Live attenuated influenza vaccines are easy to administer and might be the optimal choice for child populations in developing populations. However, further study in such populations, which might experience extended periods of influenza circulation, is warranted.

## Methods

### Study design and participants

This double-blind, placebo-controlled, parallel group, single-centre trial was done in the rural area comprising the Niakhar Demographic Surveillance System, about 110 km southeast of Dakar, Senegal. Generally healthy children aged 2–5 years were eligible if a parent was willing to provide written informed consent and was not expecting to migrate out of the area during the study period. Informed consent was obtained through a well established two-step process in accordance with established social structures and cultural norms in this population. First, the investigator held group meetings in communities of the study area to obtain village permission for study conduct. Second, a list of eligible children was constructed using demographic surveillance system data, and parents of eligible children were approached by trained study staff during the month prior to study initiation to provide them with information about the upcoming research study. Those interested in participating were given dates and locations of enrollment days at their locale. On days of enrolment, trained study staff reviewed study information with presenting parents and children, and children were enrolled consecutively as parents provided written informed consent and children were determined to be eligible to participate. Trained study physicians were present on all enrolment days. Exclusion criteria included serious active medical conditions. Acute malnutrition is common in Senegal, particularly during the typical influenza season from June through October before the harvest. Therefore, malnourished children were not specifically excluded. Eligibility criteria are listed in the appendix (p 1).

Ethics review was provided by the National Ethics Committee for Health Research (Senegal Ministry of Health and Social Welfare) and Western Institutional Review Board. The study was done in accordance with the principles of the Declaration of Helsinki (2008) and in compliance with Good Clinical Practice guidelines.

### Randomisation and masking

Participants were randomly allocated (2:1) to receive the live attenuated influenza vaccine or placebo. The allocation sequence was computer-generated by PATH with block sizes of three. The sequence was delivered to Serum Institute of India where vaccine and placebo were labelled before shipping to Senegal for use in the field. Vaccine and placebo were identical in appearance and vials containing the study products were labelled only with a clinical trial label and unique allocation numbers to preserve blinding.

### Procedures

Study products were lyophilised live attenuated influenza vaccine of 2012–13 Northern Hemisphere formulation (Nasovac-S, Serum Institute of India, Pune, India; lot 167E2002) containing A/California/7/2009 (H1N1)-like, A/Victoria/361/2011 (H3N2)-like, and B/Wisconsin/1/2010 (Yamagata lineage)-like reassortants. Matched placebo was identical to vaccine in appearance and content but was missing viral components (Serum Institute of India; lot E9001PCB). A single 0·5 mL dose of either was administered intranasally (divided evenly per nostril) to each participant using Wolfe-Tory mucosal atomiser devices. Lyophilised vaccine was stored at 2–8°C until reconstitution with diluent of ambient temperature. Administration after reconstitution was immediate.

Participant follow-up was planned from June, 2013, until December, 2013, during which participants were monitored weekly through home visits by trained field workers. On day 7 postvaccination, participants were assessed for solicited reactions and unsolicited adverse events occurring since vaccination, and on day 28 for unsolicited adverse events. Adverse events were documented by field workers using standardised data collection forms after interviews with parents at each visit. Throughout study follow-up, standardised criteria were used to identify participants with signs and symptoms of influenza. Additional criteria were used to refer participants with medically important illness to a study physician for further evaluation. These physicians provided treatment as per the standard of care in Senegal and assessed participants for study outcomes of clinical influenza, protocol-defined wheezing illness, and serious adverse events. For all participants with signs or symptoms of influenza, both a nasal swab and a pharyngeal swab specimen were collected and pooled.

A subset of participants already enrolled in the main trial were additionally consented to participate in a vaccine infectivity and extended safety subset to assess the ability of cold-adapted vaccine viruses to replicate in the nasal passages of children in this population in which ambient daily temperatures regularly exceed 40°C during the period of trial vaccinations and to provide a more detailed description of vaccine safety during the 7 days postvaccination in this low-resource population. For this analysis, both a nasal swab and a pharyngeal swab specimen were collected prevaccination and on days 2 and 4 postvaccination from all subset participants. Detailed solicited reactions and unsolicited adverse events were also collected on standard data collection forms by field workers through interview with parents/guardians.

### Outcomes

The primary efficacy endpoint was symptomatic laboratory-confirmed influenza caused by any virus type, including those not included in the vaccine, and occurring from 15 days post vaccination to the end of the study in December, 2013. Symptomatic influenza was defined as sudden onset of measured fever (>37·5°C axillary) or subjectively reported feverishness and a cough or sore throat. Laboratory confirmation (type and subtype or lineage) was defined as detection of seasonal influenza virus in a swab specimen. Specimens were tested at Senegal's National Influenza Centre Laboratory at the Institut Pasteur de Dakar for the presence of influenza virus by real-time RT-PCR using methods and reagents, including standard seasonal influenza oligonucleotide primers and probes, provided by the Influenza Division of the US Centers for Disease Control and Prevention (CDC). Additionally, although not protocol-specified, all influenza-positive specimens from symptomatic cases occurring within 1 month of vaccination were shipped to the US CDC and tested post-hoc by real-time RT-PCR using CDC's oligonucleotide primer-probe sets specific for live attenuated influenza virus A and live attenuated influenza virus B, as was done for specimens from the vaccine infectivity subset. Antigenic characterisation of about 20% of positive specimens was done at CDC. Secondary efficacy endpoints were symptomatic laboratory-confirmed influenza matched to vaccine and influenza strain-specific symptomatic influenza.

Safety endpoints included solicited local and systemic reactions (nasal congestion, runny nose, stuffy nose, ear pain, cough, sore throat, headache, fever, tachypnoea, muscle or joint pain, chills, irritability or decreased activity, and vomiting), unsolicited adverse events and serious adverse events, and protocol-defined wheezing illness. Protocol-defined wheezing illness was defined as an illness meeting physician evaluation criteria (fever >38°C axillary, tachypnoea >40 breaths per min, ear pain, seizure or convulsions, or any other condition believed to warrant physician evaluation) and characterised by a long, high-pitched whistling or musical sound on expiration heard by auscultation over the lung fields. Severity of protocol-defined wheezing illness was graded by study physicians as mild (wheezing illness without other findings associated with disease of moderate or greater severity), moderate (nasal flaring, chest in-drawing, or pulse oximetry 90–95%), severe (dyspnoea at rest causing inability to perform usual social and functional activities or pulse oximetry <90%), or life threatening (per physician's medical opinion).

Swab specimens from the vaccine infectivity subset were tested at the US CDC by real-time RT-PCR first for the presence of influenza virus using standard seasonal influenza oligonucleotide primers and probes. Positive specimens in the vaccine group were further tested for the presence of live attenuated influenza vaccine by real-time RT-PCR using oligonucleotide primers and probes designed to detect internal virus genes specific to the A/Leningrad/134/17/57 (H2N2) and B/USSR/60/69 master donor viruses used to create the cold-adapted reassortants contained in the vaccine.

### Statistical analysis

The primary objective was to estimate the efficacy of live attenuated influenza vaccine in reducing the rate of symptomatic laboratory-confirmed influenza regardless of vaccine match among children receiving live attenuated influenza vaccine. Vaccine efficacy was defined as one minus the relative rate (times 100%) of influenza in the live attenuated influenza vaccine group compared with that in the placebo group. A Cox proportional hazards model with censoring at time of first endpoint was fitted to estimate the relative rate and its 95% CI. The protocol specified that endpoints occurring within 2 weeks postvaccination were not counted to allow sufficient time for development of the immune response and to ensure that any vaccinees with non-influenza acute respiratory illness during this period would not be misclassified if vaccine virus was identified by real-time RT-PCR using seasonal influenza primer-probe sets. Secondary efficacy endpoints were similarly analysed. Primary efficacy analyses were done on a per-protocol basis and included all children who met eligibility criteria, were randomised, received one dose of study vaccine, and contributed at least one day of person-time. Supportive analyses were also done on the total vaccinated cohort of all randomised participants who received a dose of vaccine or placebo, counting endpoints occurring at any time postvaccination. Effect of previous receipt of trivalent inactivated vaccine on efficacy was explored in post-hoc analyses, as was the effect of malnutrition, with adjustment for grade of malnutrition. Grades for weight-for-age, height-for-age, and weight-for-height were based on calculated *Z* scores and categorised as mild (–2 to <–1), moderate (–3 to <–2), or severe (<–3).^11^, ^12^

Safety in the total vaccinated cohort and the extended safety subset was described as the proportion of participants in each study group experiencing reactions or events of any severity (and by severity grade) with its corresponding exact 95% CI. Fisher's exact test was used to compare proportions of reactions of any severity grade between study groups. Participant safety was also overseen by an independent safety monitoring committee convened by PATH.

Assuming 60% efficacy, 57 total primary endpoints were required to test the hypothesis that live attenuated influenza vaccine efficacy was greater than 0% with a one-sided type I error of less than 2·5% and power of at least 90%. Based on this number and assuming a 6% incidence rate and 90% evaluability, a total sample size of 1761 enrolled subjects was estimated.

Data were analysed with SAS version 9.3.

This study is registered with ClinicalTrials.gov, number NCT01854632.

### Role of the funding source

This work was funded through a Cooperative Agreement to PATH from the US Centers for Disease Control and Prevention (U01IP000476). Supplementary funding from the Bill & Melinda Gates Foundation to PATH (OPP1017334) supported statistical analyses. The findings and conclusions in this report are those of the authors and do not necessarily represent the official position of the US CDC, Gates Foundation, or PATH. All authors had full access to all the data in the study and JCV had final responsibility for the decision to submit for publication.

## Results

**Figure 1:**
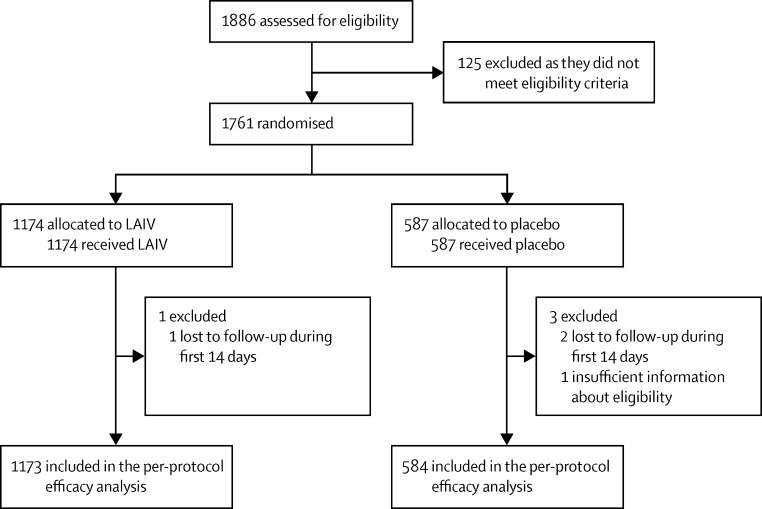
Trial profile LAIV=live attenuated influenza vaccine.

Between May 23, and July 1, 2013, 1761 children were enrolled and randomised; 1174 were allocated to receive study vaccine and 587 to receive placebo (figure 1). Nearly all (1757) contributed person-time to the primary (per-protocol) analysis of efficacy. Retention was very high, with 1722 (98%) being monitored to the end of the follow-up period, Dec 20, 2013 (table 1). Children who additionally gave consent into the vaccine infectivity and extended safety subset had their day 0 swabs taken between May 23 and June 5.

Baseline characteristics were similar in the two study groups (table 1). A substantial proportion of participants were malnourished to some extent, with 46% underweight, 48% stunted, and 36% showing wasting. Otherwise, participants had few identifiable medical issues. Only one child, in the live attenuated influenza vaccine group, had a reported history of asthma.

**Figure 2:**
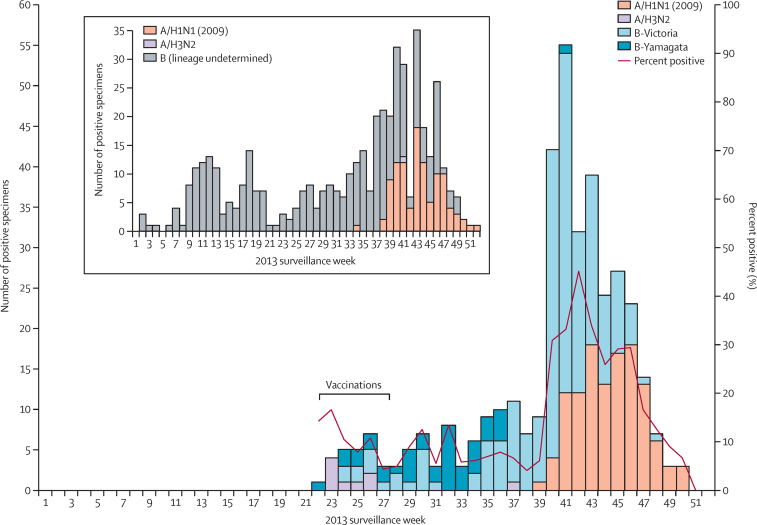
Influenza circulation in Niakhar, by type and subtype or lineage, from week 22 to week 51 during the period of the trial The graph is a stacked column chart where numbers of positive detections for each strain each week are stacked in the graph and can be visually summed. Study vaccinations occurred from week 22 to week 28. Laboratory testing data from the trial indicated that B circulation was of mixed lineage from week 22 through 35 but became almost only Victoria lineage (unmatched to vaccine) thereafter. Inset show influenza circulation in Senegal, by type and subtype, during the entire year of the trial, 2013, as measured by the Senegal National Influenza Center. Note: determination of B lineage was not standard practice for national surveillance in 2013. Data from WHO Flunet.

Type B influenza viruses of both lineages were already circulating in Senegal by the beginning of the trial (figure 2). At the trial site, the circulation of Victoria lineage B virus (unmatched to vaccine) intensified as the season progressed. Midway through the trial, H1N1pdm09 appeared and circulated extensively until the end of the study period. H3N2 circulation was negligible.

Influenza incidence among young children in this study was high. In the primary analysis, 210 endpoints of symptomatic laboratory-confirmed influenza were reached among vaccine recipients (18%) and 105 were reached among placebo recipients (18%), yielding an efficacy point estimate of 0·0% (95% CI −26·4 to 20·9%; table 2). Analyses of secondary endpoints caused by vaccine-matched strains and by specific type and subtype or lineage likewise revealed no statistical evidence for vaccine efficacy. 79 (7%) endpoints of H1N1pdm09 were noted in the vaccine group and 36 (6%) in the placebo group; 20 (2%) endpoints of vaccine-matched B in the vaccine group and 11 (2%) in the placebo group; and 115 (10%) endpoints of vaccine-mismatched B in the vaccine group and 62 (11%) in the placebo group. Only three (<1%) endpoints of H3N2, all in the vaccine group, were reached. In a post-hoc analysis of efficacy against all vaccine-matched strains using the per-protocol dataset, among children previously vaccinated with trivalent inactivated influenza vaccine (containing A/California/7/2009) in 2010 or 2011 (average age of this subgroup, 4·4 years), live attenuated influenza vaccine efficacy was −28·4% (95% CI −108·7 to 19·9) compared with 15·4% (–27·5 to 43·4) among children with no previous receipt of trivalent inactivated influenza vaccine (average age of this subgroup, 3·5 years). Additional post-hoc analyses in which we stratified primary and secondary analyses by age groups or measures of malnutrition also did not show any significant efficacy for any measure (data not shown).

Detection of at least one strain of vaccine virus was confirmed in 55 (83%) of 66 live attenuated influenza vaccine recipients postvaccination (table 3). Virus detection was highest on day 2 postvaccination. Live attenuated influenza vaccine-A/H3N2 and live attenuated influenza vaccine-B viruses were detected postvaccination among 34 (52%) and 42 (66%) of recipients, respectively. Live attenuated influenza vaccine-A/H1N1pdm09 was detected among only 14 (22%) of recipients.

One child in the live attenuated influenza vaccine group experienced mild epistaxis in the 30 min postvaccination. In the 7 days postvaccination, the most common solicited events reported overall were runny nose (296; 17%), cough (172; 10%), and nasal congestion (57; 3%). Nearly all reactions were mild, and there were no significant differences between the vaccine and placebo groups in terms of the proportions experiencing reactions of any severity (table 4) or unsolicited adverse events (appendix p 2). No significant differences were noted in occurrence of solicited reactions in the extended safety subset (appendix p 4).

Nine (1%) participants experienced protocol-defined wheezing illness, with no significant differences in occurrence between vaccine groups (table 4). Serious adverse events occurred in 14 children; seven in each of the vaccine (1%) and placebo (1%) groups (appendix p 5). All serious adverse events were considered unrelated to vaccination. Two girls, both aged 2 years, died. Both had received live attenuated influenza vaccine, and causes of death were anasarca 12 days postvaccination and malnutrition 70 days postvaccination.

## Discussion

Among this low-resource paediatric population in sub-Saharan Africa, symptomatic influenza infection was common, with an overall attack rate of about 20%. Although live attenuated influenza vaccine was well tolerated, a single dose did not provide protection against symptomatic laboratory-confirmed influenza for all strains or for the predominant vaccine-matched strain, H1N1pdm09. Efficacy was also not found for influenza B strains, although attack rates were low for the vaccine-matched B lineage. There were insufficient H3N2 cases to calculate vaccine strain-specific efficacy for that subtype. Although nearly half of participants had been previously vaccinated with trivalent inactivated influenza vaccine, randomisation ensured equal distribution of this factor to study groups in this trial, and thus it should not have biased efficacy results. The study was carefully monitored throughout, and after the results were known, we did an additional audit of the site and a validation of real-time RT-PCR testing by the Senegal National Influenza Center laboratory. These revealed no concerns which would call into question the findings of this trial.

The absence of efficacy against strain-matched, laboratory-confirmed symptomatic influenza illness in this study contrasts with the findings of a similar study conducted in the same season with a single dose of Nasovac-S among children aged 2–4 years in Bangladesh.^13^ In Bangladesh, H1N1pdm09 and H3N2 were the predominant strains, with attack rates of 3·6% and 12·3% in the placebo group, respectively, and vaccine efficacy of 50·0% (95% CI 9·2–72·5) against H1N1pdm09 and 60·4% (44·8–71·6) against H3N2. The reasons for the discrepancy between efficacy estimates in these two studies are unclear. It is unlikely that vaccine potency accounted for the efficacy differences given that the same lot of lyophilised vaccine was used for both studies. In fact, potency of shelf stocks of vaccine had been assessed monthly by Serum Institute of India, and in June, 2013, 9 months after manufacture, the A/California H1N1pdm09 component had a 50% egg infectious dose (EID_50_) of 10^7·532^. Cold chain, vaccine reconstitution, and administration were carefully monitored at both sites.

Differences in previous history of influenza exposure could exist in the Senegal and Bangladesh populations. Although we do not have prevaccination serological data to test this hypothesis, in a concurrent trial of inactivated influenza vaccines that we did in the neighbouring village of Niakhar (NCT01819155), baseline serological data showed that less than 25% of children younger than 6 years had evidence of previous exposure to H1N1pdm09, as determined by haemagglutinin antibody levels of >1:10 to that strain. Most of that seropositivity was focused in the older age groups, consistent with Senegal's national surveillance data, showing extensive H1N1pdm09 circulation in early 2010, and low levels of circulation later in 2011 and in 2012, the years before this study. Thus, it is likely that most participants in this study were naive to the H1N1 strain and would not have had antibodies that could have interfered with replication of this live vaccine virus strain. Even stratification by previous vaccination with inactivated influenza vaccine 2–3 years before this study showed no significant efficacy.

Ecology of the nasopharynx or nutritional deficiencies might differentially affect the ability of the vaccine virus to infect or the recipient to mount an immune response and, therefore, the performance of vaccine in these two populations. Although post-hoc analyses stratifying efficacy by measures of malnutrition in this study population showed no effect of nutritional status, other unmeasured micronutrient deficiencies might be important. Whether the history of recent oral polio vaccine receipt in participants in Senegal could have adversely affected the performance of the live attenuated influenza vaccine is also unknown. Previously, a study of oral polio vaccine given concomitantly with the Ann Arbor-based live attenuated influenza vaccine showed no effect on antibody responses as determined by haemagglutination inhibition assay.^14^ However, no studies have determined the effects on the immunogenicity of live attenuated influenza vaccine with oral polio vaccine use in the prior 30 days or using alternative measures of immune response.

In this study, vaccine virus of at least one strain was detected in 80% of live attenuated influenza vaccine recipients 2–4 days after administration. Since the mucociliary clearance rate is minutes, not days,^15^ we believe that our results are evidence of viral replication and that vaccine was viable at the time of administration in most recipients. However, detection of H1N1pdm09 was much lower than the other two vaccine strains. Although this low detection rate in Senegal stands in contrast to studies in Russia^16^ and Bangladesh^17^ with monovalent and trivalent formulations, respectively, where no H1N1pdm09 could be detected in nasal swabs or washes postvaccination, one study in the USA with initial trivalent live attenuated influenza vaccine containing A/California (H1N1) did find a substantial proportion of children (9/13) shed H1N1pdm09 after receipt of one dose.^18^ As mentioned earlier, differences in exposure history might exist between the Senegal and Bangladesh populations, and national surveillance data indicate that H1N1pdm09 circulation has been limited since the first wave of the pandemic in Senegal in early 2010. Prior immunity to vaccine strains has been shown to reduce live attenuated influenza vaccine viral shedding.^19^ Previous receipt of influenza vaccine has also been shown to inhibit shedding,^18^ although in that study previous vaccination was only 1 month prior and previous receipt of live attenuated influenza vaccine reduced shedding more than previous receipt of inactivated vaccine. Additionally, the earlier Bangladesh safety study used an earlier formulation of trivalent vaccine, and vaccine strains can differently interfere with each other depending on the formulation.^20^ Nonetheless, we are unable to explain why we were able to detect H1N1pdm09 vaccine virus postvaccination in about a fifth of recipients and yet measure no efficacy.

Although we assessed infectivity of vaccine virus, a major limitation of this study is its absence of immunological measurements. Standard immunogenicity measures following receipt of live attenuated influenza vaccine have lacked correlation with efficacy, and clinical efficacy studies are the standard for licensure and vaccine policy determination.^21^, ^22^ One study described cell-mediated immunity as determined by ELISPOT assays that measure γ interferon as correlating with protection following live attenuated influenza vaccine in children; however, the results have not been corroborated and this measurement is difficult in remote field settings and among children.^23^ A recent study of children aged 2–9 years further highlights the difficulty in identifying the immunological basis for protection by live attenuated influenza vaccine;^24^ the study could not identify such a mechanism in a live attenuated influenza vaccine challenge model. Regardless, had we done mucosal or serum immunologic assays, we might at least have been able to confirm that vaccinees were responding immunologically in some way to live attenuated influenza vaccine. Another limitation is that we did not assess the protection of two doses of this vaccine. Live attenuated influenza vaccines based on Ann Arbor master donor viruses (FluMist/Fluenz) are licensed beginning at 2 years of age, and two doses are recommended for children receiving influenza vaccine for the first time.^25^ The Russian-derived vaccines are licensed for single dose administration. Although comparative studies of one versus two doses of live attenuated influenza vaccines based on Russian-derived master donor viruses have not been done, studies of the Ann Arbor-based vaccines suggest that efficacy can be achieved after one dose, and might be improved by a second dose.^8^, ^26^, ^27^ However, an observational study in a broadly aged population in India found that Serum Institute of India's monovalent H1N1pdm09 vaccine had high effectiveness with only one dose.^28^ Although only half of the young children in this study in Senegal were influenza vaccine naive, whether a second dose of Nasovac-S could have provided efficacy to those either naive or previously exposed to influenza vaccine is uncertain.

Several observational studies of FluMist in the USA in 2013–14 reported reduced effectiveness against the current H1N1pdm09 among young children aged 2–8 years, with effectiveness against B preserved.^29^ Unfortunately, in view of the absence of sufficient circulating H3N2 and matched B-lineage in Senegal, we cannot determine whether the absence of efficacy in this study was strain-specific or more generalised. One hypothesis for the US findings in 2013–14 is that the H1N1pdm09 strain used for vaccine manufacture might not have been optimal because it contained a glutamic acid at position 47 (E47) in the haemagglutinin stalk that made it less thermally stable and infectious in ferrets than A/H1N1 (2009) circulating globally today.^30^ In early 2015, the manufacturer of FluMist reported that it would replace the A/California (H1N1) strain with an antigenically similar strain with a more stable haemagglutinin.^29^ However, the US CDC's effectiveness study for 2015–16, a season when H1N1pdm09 predominated,^31^ again showed no effectiveness of live attenuated influenza vaccine in mid-year analyses, leading the US Advisory Committee on Immunization Practices to recommend that the live attenuated influenza vaccine not be used in the USA for 2016–17.^32^ Confusing the issue further is that findings in other studies, other countries, and in other years have shown variable levels of effectiveness of live attenuated influenza vaccine against H1N1pdm09.^33^ Serum Institute of India's live attenuated influenza vaccine used in Senegal and the parallel Bangladesh trial^13^ also contained the E47 residue, so it is unclear how this contributed to the finding of no efficacy against H1N1pdm09 in Senegal given that efficacy was demonstrated with this vaccine in the Bangladesh trial. Finally, although standard antigenic techniques indicated circulating H1N1pdm09 was matched to vaccine, we did not sequence isolates; however, a study in Canada showed that effectiveness was preserved for current sequence changes hypothesised to result in reduced match to vaccine.^34^

The vaccine was safe and well tolerated, with fever and systemic symptoms being uncommon in the 7 days after vaccination. Local symptoms, including nasal congestion, runny nose, and cough, were common among both vaccine and placebo recipients, with no imbalances between groups. These safety results are similar to those reported in Bangladesh during both a phase 2 study and the aforementioned efficacy trial there.^13^, ^35^ In view of the association of wheezing and admissions to hospital noted among children younger than 2 years who were vaccinated with FluMist,^36^ we carefully monitored participants for wheezing illness using a standardised definition. Incidence of wheezing was overall low in this population, and we did not observe differences between the vaccine and placebo groups. These safety findings are reassuring, particularly because prelicensure trials in Russia pre-dated the FluMist finding and were not designed to identify wheezing illness as a solicited event.

Our study corroborates several years of influenza surveillance in this rural community in Senegal, which show that influenza illness is common in young children.^9^, ^37^, ^38^, ^39^ Furthermore, in most years in this tropical setting, multiple strains circulate and for extended periods. During the period of this study, vaccine-mismatched influenza B was the predominant circulating strain, supporting the need to further investigate the use of quadrivalent vaccines containing both B lineages that are already available in many parts of the world. Likewise, two doses of live attenuated influenza vaccine might be necessary for young children in Senegal and elsewhere, recognising that a two-dose schedule would be costlier and less feasible in low-resource areas of the world.

The Senegal trial of live attenuated influenza vaccine and its Bangladesh companion contribute essential data for the protection afforded to children by live attenuated influenza vaccines, especially in low-resource settings. Currently, active global discussion on the effectiveness of live attenuated influenza vaccines, especially for the current H1N1pdm09 strain, is ongoing and policy makers are basing decisions on differing results from an array of observational studies in the USA, Canada, Europe, and elsewhere. Although the Bangladesh efficacy trial provides confidence that this live attenuated influenza vaccine from India can provide protection with a single dose, our findings from Senegal are in line with recent US CDC studies in which live attenuated influenza vaccine failed to provide protection against H1N1pdm09. Further studies are needed to assess whether our findings are specific to the H1N1pdm09 strain and whether two doses might be efficacious, and to better understand the performance of live attenuated influenza vaccines in diverse paediatric populations for whom the burden of respiratory disease remains unacceptably high.

For **WHO Flunet data** see http://www.who.int/influenza/gisrs_laboratory/flunet/charts/en/

## Figures and Tables

**Table 1:** Participant completion rates and baseline characteristics

	**Live attenuated influenza vaccine (n=1174)**	**Placebo (n=587)**
**Study completion**		
Followed up to Dec 20, 2013	1148 (98%)	574 (98%)
Lost to follow-up	10 (1%)	7 (1%)
Temporary migration	14 (1%)	6 (1%)
Died	2 (<1%)	0
Age, months	47·7 (13·2)	47·3 (13·5)
**Age group (years)**		
2–<3	278 (24%)	150 (26%)
3–<4	310 (26%)	157 (27%)
4–<5	323 (28%)	145 (25%)
5–<6	263 (22%)	135 (23%)
**Sex**		
Male	610 (52%)	297 (51%)
Female	564 (48%)	290 (49%)
**Height and weight**		
Height (cm)	98·2 (9·5)	98·0 (9·4)
Weight (kg)	14·4 (2·7)	14·3 (2·8)
Underweight: weight-for-age malnutrition grade*		
None	641 (55%)	319 (54%)
Mild	333 (28%)	166 (28%)
Moderate	162 (14%)	75 (13%)
Severe	38 (3%)	27 (5%)
Stunting: height-for-age malnutrition grade*		
None	608 (52%)	311 (53%)
Mild	360 (31%)	176 (30%)
Moderate	153 (13%)	72 (12%)
Severe	53 (5%)	28 (5%)
Wasting: weight-for-height malnutrition grade*		
None	604 (51%)	289 (49%)
Mild	192 (16%)	98 (17%)
Moderate	98 (8%)	54 (9%)
Severe	41 (4%)	21 (4%)
Unknown†	239 (20%)	125 (21%)
History of chronic illness, including asthma or wheezing illness‡	1 (<1%)	0
**Receipt of trivalent inactivated influenza vaccine in previous field trials at site**		
Never	580 (49%)	285 (49%)
In mid-2009 (containing A/Brisbane/59/2007 [H1N1]-like virus)	65 (6%)	24 (4%)
In mid-2010 or mid-2011 (containing A/California/7/2009 [H1N1]-like virus)	512 (44%)	272 (46%)
Previous receipt could not be determined	17 (1%)	6 (1%)
**Receipt of oral polio vaccine in past 30 days**		
Yes	817 (70%)	413 (70%)
No	357 (30%)	174 (30%)

Data are n (%) or mean (SD).

* Based on *Z* score: mild (−2 to <–1), moderate (−3 to <–2), severe (<–3).

† Weight for height *Z* score cannot be calculated for children 60 months or older and so is categorised as unknown.

‡ One child in the live attenuated influenza vaccine group reported a history of asthma.

**Table 2:** Symptomatic laboratory-confirmed influenza outcomes among children aged 2–5 years in Senegal receiving live-attenuated influenza vaccine or placebo and estimated vaccine efficacy

	**Per-protocol population**			**Total vaccinated cohort**		
	Live attenuated influenza vaccine (n=1173)	Placebo (n=584)	Vaccine efficacy (95% CI)*	Live attenuated influenza vaccine (n=1174)	Placebo (n=587)	Vaccine efficacy (95% CI)*
**Primary virological endpoint**						
All strains*	210 (18%)	105 (18%)	0·0% (−26·4 to 20·9)	225 (19%)	107 (18%)	−6·5% (−34·1 to 15·4)
**Secondary virological endpoint**						
All vaccine-matched strains†	100 (9%)	47 (8%)	−6·1% (−50·0 to 25·0)	114 (10%)	47 (8%)	−22·6% (−72·2 to 12·7)
H1N1	79 (7%)	36 (6%)	−9·7% (−62·6 to 26·1)	79 (7%)	36 (6%)	−9·9% (−62·9 to 25·9)
H3N2‡	3 (<1%)	0	..	10 (1%)	0	..
B (Yamagata lineage, matched to vaccine)	20 (2%)	11 (2%)	9·5% (−88·9 to 56·6)	28 (2%)	11 (2%)	−27·7% (−156·5 to 36·4)
B (Victoria lineage, unmatched to vaccine)	115 (10%)	62 (11%)	7·3% (−26·3 to 31·9)	118 (10%)	64 (11%)	7·7% (−25·1 to 31·9)

Data are n (%).

* 1 minus the relative rate × 100%; relative rate was estimated using a Cox proportional hazards model.

† Only the first laboratory-confirmed influenza-like illness occurring more than 14 days postvaccination was counted in the all strains and all vaccine-matched strains analyses.

‡ H3N2: none of the three live attenuated influenza vaccine cases have been tested for live attenuated influenza vaccine *vs* wild-type virus. One case occurred 15 days postvaccination and the other two cases, one of which could not be confirmed by the WHO Collaborating Center at the US CDC as H3N2, occurred more than 100 days postvaccination. The seven additional H3N2 cases added in the total vaccinated cohort occurred during the 14-day postvaccination period; six of these were confirmed to be live attenuated influenza vaccine type influenza A.

**Table 3:** Detection of vaccine virus on day 2 and day 4 postvaccination among vaccine recipients in the vaccine infectivity subset

	**Day 2**	Day 4*	**Either day**
LAIV-A/H1N1	12/65 (19%)	3/66 (5%)	14/65 (22%)
LAIV-A/H3N2	31/65 (48%)	18/66 (27%)	34/65 (52%)
LAIV-B	34/65† (52%)	28/65 (43%)	42/64 (66%)
Any	48/65 (74%)	39/66 (59%)	55/66 (83%)

Data are n/total n (%). Specimens test-inconclusive for a particular strain were removed from the denominator of the respective table cell, except for the Any row where the specimen is counted in the denominator if it was test-positive for one of the other three vaccine strains. LAIV=live attenuated influenza vaccine.

* On day 4, three specimens had LAIV-A detected but subtype was undetermined.

† On day 2, four specimens were positive using the seasonal influenza B primers but negative using the LAIV-type B primers. On day 4, one of these four was then negative using both seasonal and LAIV-B primers, and the other three were then positive using both seasonal and LAIV-B primers. Only one participant in the placebo group (n=34) was ever positive for influenza, with only that participant's day 0 specimen testing positive for seasonal influenza B only.

**Table 4:** Local and systemic reactions in the first 7 days after vaccination and protocol-defined wheezing illness occurring throughout the trial, total vaccinated cohort

	**Live attenuated influenza vaccine (n=1171)**				**Placebo (n=587)**			
	Mild	Moderate	Severe	All*	Mild	Moderate	Severe	All*
**Local and systemic reactions in first 7 days following vaccination**								
Fever (measured ≥38°C)	2 (0·2%)	2 (0·2%)	1 (0·1%)	5 (0·4%)	1 (0·2%)	3 (0·5%)	0 (0·0%)	4 (0·7%)
Nasal congestion	41 (3·5%)	0 (0·0%)	0 (0·0%)	41 (3·5%)	16 (2·7%)	0 (0·0%)	0 (0·0%)	16 (2·7%)
Runny nose	199 (17·0%)	2 (0·2%)	0 (0·0%)	201 (17·1%)	95 (16·2%)	0 (0·0%)	0 (0·0%)	95 (16·2%)
Stuffy nose	18 (1·5%)	0 (0·0%)	0 (0·0%)	18 (1·5%)	9 (1·5%)	0 (0·0%)	0 (0·0%)	9 (1·5%)
Cough	110 (9·4%)	4 (0·3%)	0 (0·0%)	114 (9·7%)	58 (9·9%)	0 (0·0%)	0 (0·0%)	58 (9·9%)
Sore throat	4 (0·3%)	0 (0·0%)	0 (0·0%)	4 (0·3%)	4 (0·7%)	1 (0·2%)	0 (0·0%)	5 (0·9%)
Ear pain	6 (0·5%)	1 (0·1%)	0 (0·0%)	7 (0·6%)	2 (0·3%)	0 (0·0%)	0 (0·0%)	2 (0·3%)
Headache	14 (1·2%)	0 (0·0%)	0 (0·0%)	14 (1·2%)	7 (1·2%)	0 (0·0%)	0 (0·0%)	7 (1·2%)
Vomiting	11 (0·9%)	1 (0·1%)	0 (0·0%)	12 (1·0%)	6 (1·0%)	0 (0·0%)	0 (0·0%)	6 (1·0%)
Chills	3 (0·3%)	0 (0·0%)	0 (0·0%)	3 (0·3%)	1 (0·2%)	0 (0·0%)	0 (0·0%)	1 (0·2%)
Irritability/decreased activity	14 (1·2%)	2 (0·2%)	0 (0·0%)	16 (1·4%)	7 (1·2%)	1 (0·2%)	0 (0·0%)	8 (1·4%)
Muscle/joint pain	3 (0·3%)	0 (0·0%)	0 (0·0%)	3 (0·3%)	1 (0·2%)	0 (0·0%)	0 (0·0%)	1 (0·2%)
Tachypnoea (≥40 breaths per min)	1 (0·1%)	0 (0·0%)	0 (0·0%)	1 (0·1%)	0 (0·0%)	1 (0·2%)	0 (0·0%)	1 (0·2%)
**Protocol-defined wheezing illness by study period**								
Days 0–7	0 (0·0%)	0 (0·0%)	0 (0·0%)	0 (0·0%)	0 (0·0%)	0 (0·0%)	0 (0·0%)	0 (0·0%)
Days 8–42	0 (0·0%)	0 (0·0%)	0 (0·0%)	0 (0·0%)	1 (0·2%)	0 (0·0%)	0 (0·0%)	1 (0·2%)
Day 43–6+ months	3 (0·3%)	1 (0·1%)	0 (0·0%)	4 (0·3%)	2 (0·3%)	2 (0·3%)	0 (0·0%)	4 (0·7%)
Anytime	3 (0·3%)	1 (0·1%)	0 (0·0%)	4 (0·3%)	3 (0·5%)	2 (0·3%)	0 (0·0%)	5 (0·9%)

* No significant differences using Fisher's exact test (two-sided p value was never <0·05) between live attenuated influenza vaccine and placebo for events of any severity.
