# Supplementary material for: Efficacy of a Russian-backbone live attenuated influenza vaccine among children in Senegal: a randomised, double-blind, placebo-controlled trial
Source: Lancet Glob Health. 2016 Oct 13;4(12):e955–65. doi: 10.1016/S2214-109X(16)30201-7 (PMC5118222; doi:10.1016/S2214-109X(16)30201-7)
Supplement: Supplementary appendix [file mmc1.pdf]

THE LANCET Global Health

Supplementary appendix

This appendix formed part of the original submission and has been peer reviewed. We post it as supplied by the authors.

Supplement to: Victor JC, Lewis KDC, Diallo A, et al. Efficacy of a Russian-backbone live attenuated influenza vaccine among children in Senegal: a randomised, double-blind, placebo-controlled trial. *Lancet Glob Health* 2016; published online Oct 13. http://dx.doi.org/10.1016/S2214-109X(16)30201-7.

Supplementary Table 1: Eligibility Criteria

Inclusion Criteria	Exclusion Criteria	Temporary Contraindications*
Healthy male or female child at least 24 months of age and no older than 71 months of age at the time of study vaccination.	Serious, active, medical condition, including:<br>chronic disease of any body system; chronic infections such as tuberculosis; genetic disorders, such as Down's syndrome, or other cytogenetic disorder known; or suspected disease of the immune system of any kind.	Acute illness accompanied by a body temperature of 37.5°C or above (axillary measurement) within 14 days of enrollment visit.
A child whose parent or guardian's primary residence, at the time of study vaccinations, is within the Niakhar DSS and who intends to be present in the area for the duration of the trial.	History of documented hypersensitivity to eggs or other components of the vaccine (including gelatin, sorbitol, lactalbumin and chicken protein), or with life-threatening reactions to previous influenza vaccinations.	Any acute respiratory infection within 14 days of enrollment visit.
A child whose parent or legal guardian is willing to provide written informed consent prior to the participant's study vaccination.	History of Guillain-Barré syndrome.	Any illness accompanied by active wheezing within 14 days of enrollment visit.
	Receipt of immunosuppressive agents, including systemic corticosteroids, during the month before planned study vaccination.	
	Receipt of aspirin therapy or aspirin-containing therapy within the two weeks before planned study vaccination.	
	History of any severe allergic reaction with generalized urticarial, angioedema, or anaphylaxis.	
	Receipt of an influenza vaccine within the past 12 months.	
	Has any condition determined by investigator as likely to interfere with evaluation of the vaccine or be a significant potential health risk to the child or make it unlikely that the child would complete the study.	

*Enrollment and administration of LAIV or placebo postponed until at least 14 days after recovery.

Supplementary Table 2: Proportion of participants in each study arm with non-serious unsolicited adverse events by MedDRA System Organ Class, Preferred Term, and severity, Total Vaccinated Cohort.*

	LAIV n=1174					Placebo n=587				
	Mild	Moderate	Severe	Missing	Any	Mild	Moderate	Severe	Missing	Any
	number (%)									
**Any unsolicited Adverse Event**										
**total**	**37 (3·2)**	**16 (1·4)**	**3 (0·3)**	**0**	**56 (4·8)**	**17 (2·9)**	**14 (2·4)**	**1 (0·2)**	**1 (0·2)**	**33 (5·6)**
**Ear and labyrinth disorders**										
ear pain	0	1 (0·1)	0	0	1 (0·1)	0	0	0	0	0
mostoiditis	0	1 (0·1)	0	0	1 (0·1)	0	1 (0·2)	0	0	1 (0·2)
otitis media	2 (0·2)	2 (0·2)	0	0	4 (0·3)	1 (0·2)	0	0	0	1 (0·2)
**total**	**2 (0·2)**	**4 (0·3)**	**0**	**0**	**6 (0·5)**	**1 (0·2)**	**1 (0·2)**	**0**	**0**	**2 (0·3)**
**Gastrointestinal disorders**										
abdominal pain	8 (0·7)	0	0	0	8 (0·7)	6 (1·0)	0	0	0	6 (1·0)
diarrhoea	20 (1·7)	1 (0·1)	0	0	21 (1·8)	5 (0·9)	0	0	1 (0·2)	6 (1·0)
enteritis	1 (0·1)	1 (0·1)	0	0	2 (0·2)	0	0	0	0	0
gastroenteritis	1 (0·1)	0	0	0	1 (0·1)	0	1 (0·2)	0	0	1 (0·2)
vomiting*	1 (0·1)	0	0	0	1 (0·1)	1 (0·2)	0	0	0	1 (0·2)
**total**	**26 (2·2)**	**2 (0·2)**	**0**	**0**	**28 (2·4)**	**11 (1·9)**	**1 (0·2)**	**0**	**1 (0·2)**	**13 (2·2)**
**General disorders and administration site conditions**										
malaise	0	1 (0·1)	0	0	1 (0·1)	0	0	0	0	0
pyrexia	1 (0·1)	0	0	0	1 (0·1)	0	2 (0·3)	0	0	2 (0·3)
**total**	**1 (0·1)**	**1 (0·1)**	**0**	**0**	**2 (0·2)**	**0**	**2 (0·3)**	**0**	**0**	**2 (0·3)**
**Infections and infestations**										
abscess	1 (0·1)	0	0	0	1 (0·1)	0	0	0	0	0
dysentery	0	1 (0·1)	0	0	1 (0·1)	0	1 (0·2)	0	0	1 (0·2)
ear infection	0	1 (0·1)	0	0	1 (0·1)	0	0	0	0	0
infection	2 (0·2)	1 (0·1)	1 (0·1)	0	4 (0·3)	0	0	1 (0·2)	0	1 (0·2)

mumps	3 (0·3)	1 (0·1)	0	0	4 (0·3)	0	1 (0·2)	0	0	1 (0·2)
**total**	**5 (0·4)**	**4 (0·3)**	**1 (0·1)**	**0**	**10 (0·9)**	**0**	**2 (0·3)**	**1 (0·2)**	**0**	**3 (0·5)**
**Injury, poisoning, and procedural complications**										
skin wound	1 (0·1)	0	0	0	1 (0·1)	0	0	0	0	0
wound	2 (0·2)	0	0	0	2 (0·2)	0	0	0	0	0
**total**	**3 (0·3)**	**0**	**0**	**0**	**3 (0·3)**	**0**	**0**	**0**	**0**	**0**
**Nervous system disorders**										
somnolence	0	0	0	0	0	1 (0·2)	0	0	0	1 (0·2)
**total**	**0**	**0**	**0**	**0**	**0**	**1 (0·2)**	**0**	**0**	**0**	**1 (0·2)**
**Respiratory, thoracic and mediastinal disorders**										
brochiolitis	2 (0·2)	2 (0·2)	0	0	4 (0·3)	0	0	0	0	0
nasopharyngitis	2 (0·2)	6 (0·5)	0	0	8 (0·7)	3 (0·5)	0	0	0	3 (0·5)
oropharyngeal pain	0	0	0	0	0	0	1 (0·2)	0	0	1 (0·2)
pneumonia	2 (0·2)	0	2 (0·2)	0	4 (0·3)	3 (0·5)	5 (0·9)	0	0	8 (1·4)
pulmonary congestion	0	1 (0·1)	0	0	1 (0·1)	0	0	0	0	0
runny nose*	3 (0·3)	0	0	0	3 (0·3)	0	0	0	0	0
upper respiratory tract infection	0	0	0	0	0	1 (0·2)	3 (0·5)	0	0	4 (0·7)
**total**	**8 (0·7)**	**7 (0·6)**	**2 (0·2)**	**0**	**17 (1·4)**	**7 (1·2)**	**9 (1·5)**	**0**	**0**	**16 (2·7)**

*Only non-serious adverse events occurring within the first 31 days post-vaccination are summarized. Participants are counted only once based on maximum severity, but can contribute to more than one category.

Supplementary Table 3. Local and systemic reactions in the first 7 days following vaccination, extended safety subset*

	LAIV (N=68)				Placebo (N=32)			
	Mild	Moderate	Severe	All**	Mild	Moderate	Severe	All**
	number (%)							
				(95% CI, %)				(95% CI, %)
**Fever (measured >= 38°C)**	1 (1·5)	1 (1·5)	0 (0·0)	2 (2·9)<br>(0·4 to 10·2)	0 (0·0)	0 (0·0)	0 (0·0)	0 (0·0)<br>(0·0 to 10·9)
**Nasal congestion**	3 (4·4)	0 (0·0)	0 (0·0)	3 (4·4)<br>(0·9 to 12·4)	0 (0·0)	0 (0·0)	0 (0·0)	0 (0·0)<br>(0·0 to 10·9)
**Runny nose**	13 (19·1)	0 (0·0)	0 (0·0)	13 (19·1)<br>(10·6 to 30·5)	4 (12·5)	0 (0·0)	0 (0·0)	4 (12·5)<br>(3·5 to 29·0)
**Stuffy nose**	1 (1·5)	0 (0·0)	0 (0·0)	1 (1·5)<br>(0·0 to 7·9)	2 (6·3)	0 (0·0)	0 (0·0)	2 (6·3)<br>(0·8 to 20·8)
**Cough**	7 (10·3)	0 (0·0)	0 (0·0)	7 (10·3)<br>(4·2 to 20·1)	3 (9·4)	0 (0·0)	0 (0·0)	3 (9·4)<br>(2·0 to 25·0)
**Sore throat**	0 (0·0)	0 (0·0)	0 (0·0)	0 (0·0)<br>(0·0 to 5·3)	0 (0·0)	0 (0·0)	0 (0·0)	0 (0·0)<br>(0·0 to 10·9)
**Ear Pain**	0 (0·0)	0 (0·0)	0 (0·0)	0 (0·0)<br>(0·0 to 5·3)	0 (0·0)	0 (0·0)	0 (0·0)	0 (0·0)<br>(0·0 to 10·9)
**Headache**	2 (2·9)	0 (0·0)	0 (0·0)	2 (2·9)<br>(0·4 to 10·2)	0 (0·0)	0 (0·0)	0 (0·0)	0 (0·0)<br>(0·0 to 10·9)
**Vomiting**	0 (0·0)	1 (1·5)	0 (0·0)	1 (1·5)<br>(0·0 to 7·9)	1 (3·1)	0 (0·0)	0 (0·0)	1 (3·1)<br>(0·1 to 16·2)
**Chills**	1 (1·5)	0 (0·0)	0 (0·0)	1 (1·5)<br>(0·0 to 7·9)	0 (0·0)	0 (0·0)	0 (0·0)	0 (0·0)<br>(0·0 to 10·9)
**Irritability/ Decreased Activity**	1 (1·5)	0 (0·0)	0 (0·0)	1 (1·5)<br>(0·0 to 7·9)	0 (0·0)	0 (0·0)	0 (0·0)	0 (0·0)<br>(0·0 to 10·9)
**Muscle/joint pain**	1 (1·5)	0 (0·0)	0 (0·0)	1 (1·5)<br>(0·0 to 7·9)	0 (0·0)	0 (0·0)	0 (0·0)	0 (0·0)<br>(0·0 to 10·9)
**Tachypnea (>=40 breaths/min)**	1 (1·5)	0 (0·0)	0 (0·0)	1 (1·5)<br>(0·0 to 7·9)	0 (0·0)	0 (0·0)	0 (0·0)	0 (0·0)<br>(0·0 to 10·9)

*Participants in the extended safety subset had visits made on Days 2, 4, and 7 post-vaccination to inquire about experienced reactions.

**There were no significant differences using Fisher's exact test (2-sided p-value was never <0·05) between LAIV and placebo for events of any severity.

Supplementary Table 4: Serious Adverse Events occurring during the study

Study group/ Subject number	Event	Onset day post-vaccination	SAE criteria	Outcome
**LAIV**				
1-2009	Anasarca (caused acute glomerulonephritis as determined via verbal autopsy)	11	death	death
1-0053	Acute glomerulonephritis associated with streptococcoal infection	60	hospitalization	recovered
1-0192	Malnutrition accompanied by multiple infections	63	hospitalization	death
2-0772	Malnutrition in child of low birth-weight	85	hospitalization	recovery ongoing
1-0015	Pneumonia	125	medically important	recovered
2-0959	Tibia fracture	128	medically important	recovered
1-0172	Convulsive seizure associated with acute respiratory infection	178	hospitalization	recovered
**Placebo**				
2-0967	Burn wound infection	65	hospitalization	recovered
1-0618	Humerus fracture	68	medically important	recovered
2-1013	Abscess of knee bursa	90	hospitalization	recovered
1-0394	Pneumonia	116	medically important	recovered
2-0876	Clavicle fracture	116	medically important	recovered
3-1755	Pneumonia	138	medically important	recovered
2-0736	Humerus fracture with luxation at elbow	181	medically important	recovered
